# An elusive diagnosis: pericardial effusion and hypereosinophilia revealing seronegative eosinophilic granulomatosis with polyangitis: a case report

**DOI:** 10.1097/MS9.0000000000005667

**Published:** 2026-09-17

**Authors:** Vishal Akula, Zaid Naseer, Norman Bernstein, Aaron Schatz

**Affiliations:** a Mary Washington Healthcare Internal Medicine Resident, Fredericksburg, Virginia, USA; b Mary Washington Internal Medicine Resident, Fredericksburg, Virginia, USA; c Mary Washington Infectious Disease Attending, Fredericksburg, Virginia, USA; d Mary Washington Cardiology Attending, Fredericksburg, Virginia, USA

## Abstract

Eosinophilic granulomatosis with polyangiitis (EGPA) is a small- and medium-vessel necrotizing vasculitis characterized by asthma, eosinophilia, and multiorgan involvement. Cardiac manifestations, though uncommon, are more prevalent in antineutrophil cytoplasmic antibody (ANCA)-negative patients and carry significant morbidity. Diagnosis can be challenging due to variable presentations and the lack of a definitive test.

We present a 60-year-old woman with asthma and chronic rhinosinusitis who developed progressive dyspnea and was found to have a large pericardial effusion with tamponade physiology, bilateral pulmonary infiltrates, and marked eosinophilia. She underwent an urgent pericardial window and a lung wedge resection. Lung biopsy revealed eosinophilic vasculitis and eosinophilic pneumonia. Extensive infectious and autoimmune workups, including ANCA testing, was negative. She demonstrated rapid clinical improvement following the initiation of corticosteroids. The combination of asthma, sinus disease, eosinophilia, pulmonary infiltrates, and biopsy findings met the 2022 ACR–EULAR classification criteria for EGPA. She was discharged on high-dose steroids and was later started on mepolizumab for maintenance.

This case highlights the diagnostic complexity of EGPA, particularly in ANCA-negative patients with life-threatening features such as cardiac tamponade. It underscores the importance of maintaining a broad differential in patients with systemic eosinophilia. Early recognition, timely immunosuppressive therapy, and multidisciplinary care are critical for improving outcomes and preventing severe complications.

## Introduction

Eosinophilic granulomatosis with polyangiitis (EGPA), formerly known as Churg–Strauss syndrome, is a multisystem disorder most commonly characterized by asthma, rhinitis, nasal polyps, and marked peripheral blood and tissue eosinophilia^[^1^]^. It is considered a variant within the group of diseases defined by necrotizing vasculitis affecting small- and medium-sized blood vessels. EGPA is classified as an antineutrophil cytoplasmic antibody (ANCA)-associated vasculitis. Among ANCA-associated vasculitides, EGPA is one of the rarer subtypes, with an estimated prevalence of 1–3 cases per million individuals^[^1^]^. The mean age at diagnosis ranges from 38 to 54 years, with a median of 40^[^1^]^. However, cases have been reported at both ends of the age spectrum. The condition affects males and females at similar rates.

HIGHLIGHTS

- This article explores the diagnostic pathway for **antineutrophil cytoplasmic antibody (ANCA)-negative Eosinophilic Granulomatosis with Polyangiitis (EGPA)**.
- It highlights the differences in clinical and pathological characteristics between **ANCA-positive and ANCA-negative EGPA**.
- It reviews current **treatment strategies** for EGPA, including corticosteroids and targeted biologic therapies.
- It discusses **conditions that can mimic EGPA**, emphasizing the importance of accurate differential diagnosis.

The pathogenesis of EGPA involves either eosinophil-mediated tissue damage or ANCA-induced endothelial injury. Eosinophil-driven damage is initiated by a T-helper 2 (Th2) immune response, leading to eosinophil margination^[^1^]^. Interleukins IL-3 and IL-5, produced by Th2 lymphocytes, regulate eosinophil maturation, release, and survival in circulation^[^1^]^. Activated eosinophils release cytotoxic proteins that contribute directly to tissue damage. Histologically, EGPA is characterized by eosinophilic infiltrates within blood vessel walls and extravascular tissues.

Although EGPA is considered an ANCA-associated vasculitis, approximately 60% of patients are ANCA-negative. Among the ANCA-positive group, 35% are myeloperoxidase (MPO)-positive, and 5% are proteinase 3 (PR3)-positive^[^1^]^. Studies have demonstrated distinct clinical patterns based on ANCA status: ANCA-positive patients are more likely to present with constitutional symptoms, mononeuritis multiplex, and pulmonary capillaritis, whereas ANCA-negative individuals are more prone to eosinophilic pulmonary infiltrates and cardiac involvement^[^2,3^]^.

Diagnosing EGPA remains challenging due to the lack of a definitive diagnostic test. Diagnosis relies on a combination of clinical features, histopathology, and laboratory investigations. In 2022, the American College of Rheumatology (ACR) and the European Alliance of Associations for Rheumatology (EULAR) developed classification criteria with a scoring system for EGPA. A cumulative score of six or more yields an 85% sensitivity and 99% specificity for the diagnosis^[^4^]^.

This case report describes a patient with multiorgan involvement at presentation, which ultimately led to the diagnosis of EGPA.

## Case

A 60-year-old female with a history of asthma and chronic rhinosinusitis presented with subacute, progressive dyspnea and a productive cough. She had multiple prior hospitalizations for presumed asthma exacerbations and had received short- and long-acting beta-agonists, antimuscarinic agents, inhaled corticosteroids, and systemic corticosteroids as clinically indicated. She denied any history of smoking or recent travel. She worked as a cashier in a pet store but reported no significant direct exposure to animals.

On admission, laboratory evaluation revealed marked eosinophilia with an absolute eosinophil count of 8.54 K/μL (Table 1), along with a significantly elevated N-terminal pro–B-type natriuretic peptide level of 8690 pg/mL. Her comprehensive metabolic panel was within normal limits, aside from a mild elevation in alkaline phosphatase (Table 2). Computed tomography of the chest demonstrated a moderate pericardial effusion and bilateral patchy pulmonary infiltrates (Figs 1 and 2). Transthoracic echocardiography confirmed a large pericardial effusion with tamponade physiology, including right atrial and right ventricular diastolic collapse.

**Figure 1.**
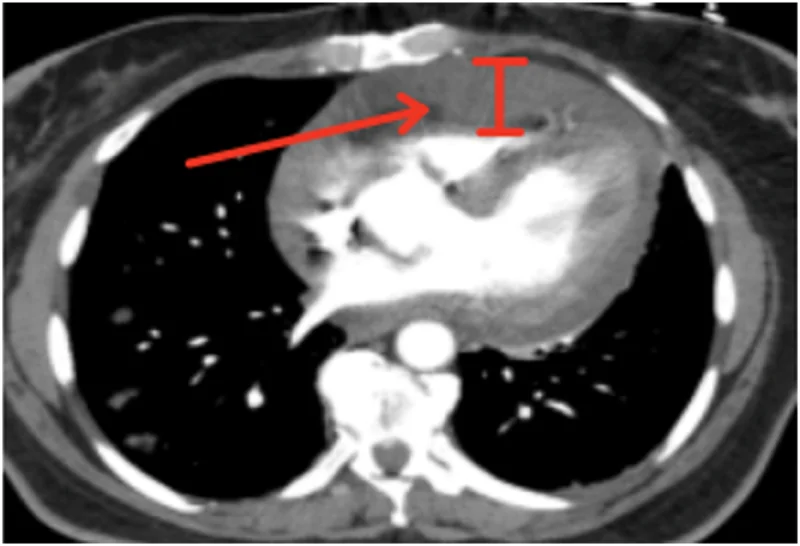
Axial computed tomography view of a moderate-volume pericardial effusion (red arrow).

**Figure 2.**
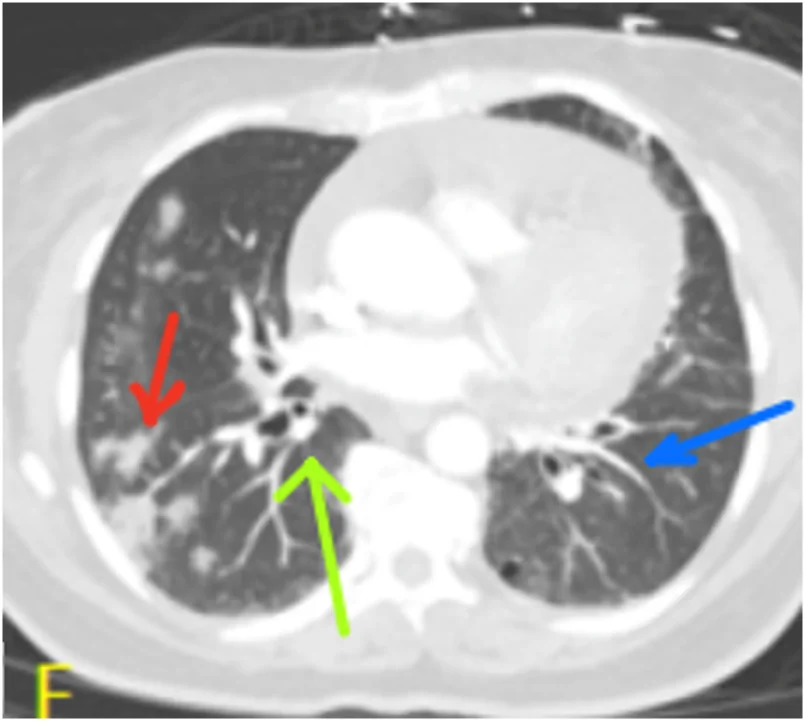
Axial computed tomography lung view showing numerous scattered, ill-defined ground-glass opacities (red arrow) and diffuse peribronchovascular thickening (blue arrow), with prominent mediastinal and hilar lymph nodes (green arrow), including a subcarinal lymph node measuring 1.3 cm.

**Table 1:** Complete blood cell count.

Component	
RBC (×10^6^/µL)	3.58 (Low)
Hemoglobin (g/dL)	10.3 (Low)
Hematocrit (%)	32 (Low)
MCV (fL)	90
MCH (pg)	29
MCHC (g/dL)	32
Platelets (×10^3^/µL)	339
RDW-SD (fL)	47.9 (High)
RDW-CV (%)	14.6
MPV (fL)	9.1
nRBC (%)	0
Neutrophils %	23 (Low, R)
Lymphocytes %	13 (Low)
Monocytes %	1 (Low, R)
Eosinophils %	62 (High)
Basophils %	0 (R)
Immature granulocytes %	–
Neutrophils absolute (×10^3^/µL)	3.31
Lymphocytes absolute (×10^3^/µL)	1.79
Monocytes absolute (×10^3^/µL)	0.14
Eosinophils absolute (×10^3^/µL)	8.54 (High)
Basophils absolute (×10^3^/µL)	0.00
Immature granulocytes absolute (×10^3^/µL)	–
Differential type	Manual

**Table 2:** Complete metabolic panel.

**Parameter**	
Glucose (mg/dL)	120 (High)
BUN (mg/dL)	21
Creatinine (mg/dL)	1.1
Sodium (mmol/L)	143
Potassium (mmol/L)	4.4
Chloride (mmol/L)	105
CO_2_ (mmol/L)	28
Calcium (mg/dL)	9.6
Total protein (g/dL)	8.7 (High)
Albumin (g/dL)	3.7
AST (U/L)	20
Alkaline phosphatase (U/L)	161 (High)
ALT (U/L)	16
Total bilirubin (mg/dL)	0.5

Pericardiocentesis was unsuccessful, necessitating urgent video-assisted thoracoscopic surgery (VATS) with the creation of a pericardial window and a lung wedge resection. Cytologic analysis of the pericardial fluid demonstrated reactive changes without evidence of malignancy. Histopathologic examination of the lung tissue revealed eosinophilic infiltration of vessel walls, consistent with vasculitis, as well as features of eosinophilic pneumonia (Fig. 3).

**Figure 3.**
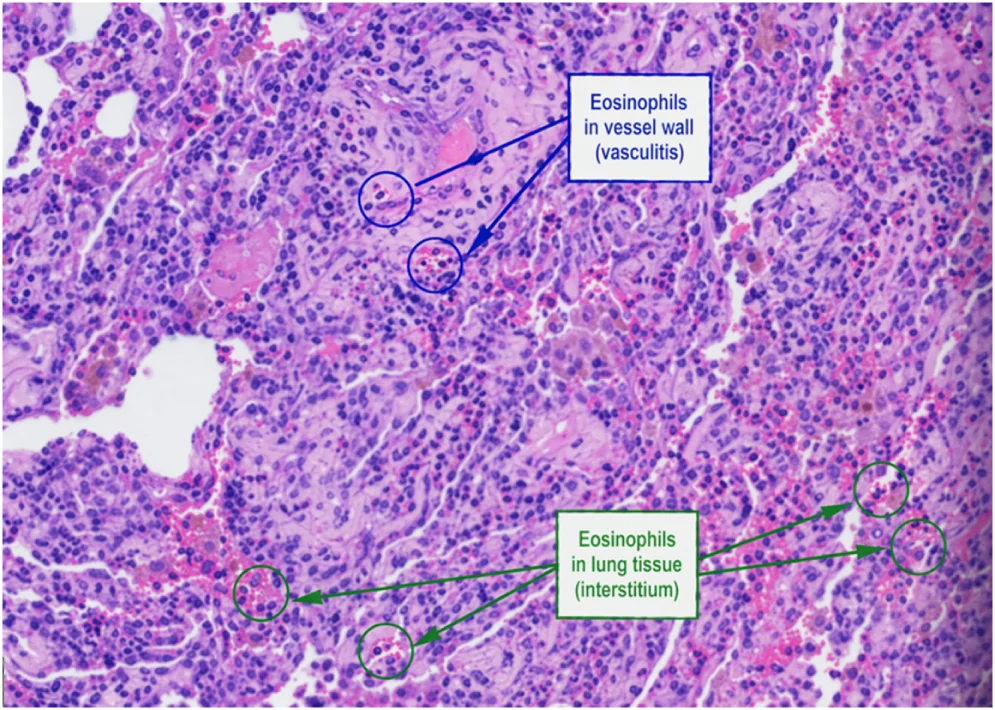
Biopsied lung tissue demonstrates eosinophilic infiltration involving both the vessel walls and the pulmonary interstitium.

Given the marked eosinophilia and systemic involvement, infectious etiologies were initially suspected. However, testing for parasites (Strongyloides, Coccidioides, Cryptococcus) and broad infectious panels, including Karius testing, returned negative results. Autoimmune workup, including ANA, ANCA, MOP antibody, PR3, was also negative. Despite antimicrobial therapy, eosinophilia persisted.

Due to ongoing multiorgan involvement and hypereosinophilia, intravenous corticosteroids were initiated, resulting in rapid clinical improvement and raising suspicion of EGPA. Although serologic markers were negative, the combination of asthma, eosinophilia, sinus disease, pulmonary infiltrates, and histologic evidence of eosinophilic vasculitis met the 2022 ACR-EULAR classification criteria for EGPA. The patient was discharged on high-dose oral steroids with follow-up with Rheumatology and Pulmonology. A subsequent sinus CT revealed sinonasal polyposis, further supporting the diagnosis of EGPA. She was later started on mepolizumab for maintenance therapy.

## Discussion

This case underscores the diagnostic complexity of EGPA, particularly in instances in which initial objective data, including autoimmune serologies, may be unremarkable. Despite this, the diagnosis can still be established based on the overall clinical picture. The patient presented with multisystem involvement, including pericardial effusion, pulmonary infiltrates, and marked peripheral eosinophilia, necessitating a broad initial differential diagnosis.

Given the degree of eosinophilia, a parasitic infection was initially considered; however, an extensive infectious workup, including HIV testing, was negative. Attention subsequently shifted toward autoimmune and malignant etiologies in the context of systemic involvement. Malignancy was ultimately excluded following tissue biopsy, which instead raised suspicion for an underlying autoimmune process.

EGPA is traditionally described as progressing through three phases:

- **Prodromal phase** – Characterized by nonspecific constitutional symptoms such as fever, weight loss, malaise, and polyarthralgias, often in association with adult-onset asthma. Upper respiratory tract involvement is common, with chronic rhinosinusitis reported in 47–93% of patients and nasal polyps in 62–77%^[^1^]^.

- **Eosinophilic phase** – Defined by peripheral eosinophilia and eosinophilic infiltration of end organs, commonly presenting with pulmonary infiltrates or eosinophilic gastrointestinal disease.
- **Vasculitic phase** – Typically occurring 3–9 years after the onset of asthma, this phase is characterized by systemic small- to medium-vessel vasculitis.

It is important to note that these phases are not always discrete and frequently overlap. In this case, the patient’s presentation reflects features of both the prodromal and eosinophilic phases, given her history of asthma and chronic rhinosinusitis, along with pulmonary infiltrates and significant peripheral eosinophilia.

The diagnosis of EGPA remains challenging due to the absence of a single definitive test and is primarily clinical in nature. The 2022 ACR/EULAR classification criteria provide a structured framework that assigns weighted points to clinical, histopathologic, and laboratory features. A cumulative score of ≥6 demonstrates a sensitivity of 85% and a specificity of 99% for EGPA. Table 3 summarizes the point allocation for each criterion.

**Table 3:** 2022 ACR – EULAR classification criteria.

Maximum eosinophil count of 1 × 10^9^ cells/L or higher	+5
Obstructive airway disease	+3
Nasal polyps	+3
Mononeuritis multiplex or motor neuropathy not due to radiculopathy	+1
Extravascular eosinophilic predominant inflammation	+2
ANCA or anti-proteinase 3-ANCA positivity	−3
Hematuria	−1

Serologic testing, particularly for antineutrophil cytoplasmic antibodies (ANCA), can support the diagnosis of EGPA; however, only approximately 30–40% of patients are ANCA-positive^[^1^]^. ANCA-negative patients typically demonstrate less small-vessel vasculitic involvement – such as purpura, renal disease, and peripheral neuropathy – but are more likely to exhibit severe asthma and cardiac manifestations^[^1^]^. This distinction is thought to reflect a predominance of tissue eosinophil–mediated pathology in ANCA-negative disease. Additionally, ANCA-negative patients often present with higher eosinophil counts, gastrointestinal involvement, and pulmonary infiltrates.

This pattern is reflected in the present case. The patient developed cardiac tamponade physiology, and pericardial fluid cytology demonstrated reactive, nonspecific changes. However, when considered in the broader clinical context of pulmonary infiltrates and marked hypereosinophilia, these findings support the working diagnosis of EGPA. Furthermore, the clinical presentation aligns with existing literature describing a higher incidence of cardiac involvement in ANCA-negative EGPA^[^1^]^.

This case is also notable for the degree of hypereosinophilia observed. Although multiple cases of ANCA-negative EGPA have been reported, the magnitude of eosinophilia in this patient appears to exceed that described in prior literature. The patient’s peak absolute eosinophil count was 8.54 K/μL, which is substantially higher than values reported in other case studies. For example, prior reports by Chen (2023), Chen (2025), and Kasama (2022) documented eosinophil counts of 3.79 K/μL, 1.13 K/μL, and 3.7 K/μL, respectively^[^5,6,7^]^. This suggests that the degree of eosinophilia in this case is among the highest reported to date^[^5,6,7^]^.

Although ANCA testing was negative in this patient, the constellation of findings – including pericardial effusion, pulmonary infiltrates, marked eosinophilia, and a history of severe asthma with frequent exacerbations – strongly supports the diagnosis of ANCA-negative EGPA. The presence of chronic rhinosinusitis and nasal polyps, identified on outpatient sinus imaging, further strengthens this diagnosis.

During hospitalization, the patient required prolonged mechanical ventilation due to excessive secretions, which evolved into a mixed allergic and non-allergic rhinitis phenotype. Post-extubation imaging confirmed nasal polyps, consistent with EGPA-related upper airway involvement and contributing to the overall diagnostic assessment.

The patient met the 2022 ACR/EULAR classification criteria for EGPA with a total score of 10 points, derived from:

Nasal polyps (+3)

Blood eosinophil count > 1000 cells/μL (+5).

Extravascular eosinophil-predominant inflammation on biopsy (+2).

Hypereosinophilic syndrome (HES) was considered in the differential diagnosis because it can closely mimic EGPA. However, this patient’s clinical features were more consistent with EGPA, particularly the presence of asthma and nasal polyps – findings that are uncommon in HES. Furthermore, histopathologic evidence of eosinophilic infiltration within the vessel walls supports a vasculitic process, which is characteristic of EGPA and rare in HES.

Importantly, the patient did not improve with broad-spectrum antibiotics and fluid resuscitation, prompting further evaluation for an inflammatory or autoimmune etiology. Given persistent eosinophilia, pulmonary infiltrates, and clinical deterioration, high-dose corticosteroid therapy was initiated. The patient demonstrated rapid clinical improvement following the initiation of intravenous methylprednisolone (80 mg every 8 hours), with a marked decline in eosinophil count from 8.54 K/μL to 3.58 K/μL and subsequently to 0.01 K/μL within 3 days. Her respiratory status improved significantly, allowing successful extubation, further supporting an inflammatory etiology.

The Five-Factor Score (FFS), developed by the French Vasculitis Study Group, has historically been used to stratify disease severity and guide treatment decisions in EGPA. More recent guidance from the ACR classifies disease as active, severe, non-severe, or in remission and provides corresponding treatment recommendations. Non-severe disease may be managed with corticosteroids and mepolizumab, whereas severe disease – particularly with cardiac, gastrointestinal, or renal involvement – typically requires additional immunosuppressive therapy such as cyclophosphamide or rituximab, in combination with corticosteroids^[^1,8,9^]^.

An FFS score of ≥1 is associated with an increased risk of relapse and mortality. In this case, the patient had an FFS score of 1 due to cardiac involvement, categorizing her disease as severe. Consistent with ACR criteria, her initial presentation was also classified as severe^[^9^]^. Accordingly, initiation of high-dose corticosteroids was appropriate. Although guideline-directed therapy would typically include the addition of cyclophosphamide or rituximab, this was not initiated due to the absence of in-house rheumatology services at the treating facility. The patient was therefore managed with high-dose corticosteroid monotherapy, with contingency plans for transfer if clinical improvement did not occur. Fortunately, her condition improved significantly without escalation of care.

Following discharge, the patient was transitioned to oral corticosteroids and was initiated on mepolizumab in the outpatient setting. At that time, her disease was considered active but non-severe, as her cardiac manifestations had resolved following surgical intervention. In accordance with ACR guidelines, mepolizumab in combination with corticosteroids is recommended in this clinical context^[^9^]^.

Prognosis in EGPA is variable, with cardiac involvement representing a major predictor of mortality and accounting for up to 50% of EGPA-related deaths^[^10,11,12,13^]^. This underscores the importance of early recognition and prompt initiation of appropriate therapy.

## Conclusion

EGPA is a rare, multisystem disorder associated with significant morbidity, making early diagnosis and management essential. In patients presenting with multisystem involvement, clinicians must maintain a broad differential diagnosis and carefully integrate clinical, laboratory, and histopathologic data. Presentations may not conform to classic patterns, and a high index of suspicion is often required. This case highlights the importance of maintaining diagnostic flexibility and pursuing a thorough evaluation, ultimately enabling timely diagnosis and effective treatment.

## Data Availability

The information included in this case report was obtained from patient medical records. Additional data are not publicly available to protect patient confidentiality.

## References

[1] Chakraborty RK Rout P. Eosinophilic granulomatosis with polyangiitis (Churg-Strauss Syndrome) [Updated 2024 Sep 19]. In: StatPearls. StatPearls Publishing; 2025. https://www.ncbi.nlm.nih.gov/books/NBK537099/30725784

[2] Yılmaz İ Tutar N Şimşek ZÖ. Clinical and serological features of eosinophilic and vasculitic phases of eosinophilic granulomatosis with poliangiitis: a case series of 15 patients. Turk Thorac J 2017;18:72–77. 10.5152/TurkThoracJ.2017.16040 PMC5783085 29404165

[3] Sablé-fourtassou R Cohen P Mahr A. Antineutrophil cytoplasmic antibodies and the Churg-Strauss syndrome. Ann Intern Med 2005;143:632–38. 10.7326/0003-4819-143-9-200511010-00006 16263885

[4] Grayson PC Ponte C Suppiah R. American college of rheumatology/European alliance of associations for rheumatology classification criteria for eosinophilic granulomatosis with polyangiitis. Ann Rheum Dis 2022;81:309–14. 10.1136/annrheumdis-2021-221794 35110334

[5] Chen Y Wan Q Liu B. ANCA-negative eosinophilic granulomatosis with polyangiitis complicated by peripheral nerve damage: a case report. Medicine (Baltimore). 2023;102:e34450. doi:10.1097/MD.0000000000034450. PMC10403021 37543822

[6] Chen J Liu J Luo M. ANCA-negative eosinophilic granulomatosis with polyangiitis presenting with duodenal stenosis: a case report. Am J Case Rep 2025;26:e951493. 10.12659/AJCR.951493 PMC12790135 41454498

[7] Kasama E Ino J Iemura F. ANCA-negative vasculitis in eosinophilic granulomatosis with polyangiitis complicated with membranous nephropathy: a case report and brief literature review. Case Rep Nephrol 2022;2022:8110940. 10.1155/2022/8110940 PMC9106512 35573079

[8] Chung SA Langford CA Maz M. American college of rheumatology/vasculitis foundation guideline for the management of antineutrophil cytoplasmic antibody-associated vasculitis. Arthritis Care Res (Hoboken) 2021;73:1088–105. 10.1002/acr.24634 PMC12344527 34235880

[9] Chung S. 2021-ACR-VF-guideline-for-management-of-ANCA-. n.d. Accessed 2 February 2026. https://vasculitisfoundation.org/wp-content/uploads/2024/01/2021-ACR-VF-Guideline-for-Management-of-ANCA-Associated-Vasculitis.pdf

[10] Lanham JG Elkon KB Pusey CD. Systemic vasculitis with asthma and eosinophilia: a clinical approach to the Churg-Strauss syndrome. Medicine (Baltimore) 1984;63:65–81. 10.1097/00005792-198403000-00001 6366453

[11] Mert A. Churg-Strauss syndrome: a case report. J Family Med Prim Care 2022;11:5656–58. 10.4103/jfmpc.jfmpc_547_21 PMC9731080 36505587

[12] Smedema JP van Paassen P van Kroonenburgh MJ. Cardiac involvement of Churg Strauss syndrome demonstrated by magnetic resonance imaging. Clin Exp Rheumatol 2004;22:S75–8. 15675140

[13] Ullah S Ali M Kyaw P. P-ANCA negative eosinophilic granulomatosis with polyangiitis. Respir Med Case Rep 2019;27:100830. 10.1016/j.rmcr.2019.100830 PMC6446216 30989049

